# Atomic Force Microscopy Methods to Measure Tumor Mechanical Properties

**DOI:** 10.3390/cancers15133285

**Published:** 2023-06-22

**Authors:** Julian Najera, Matthew R. Rosenberger, Meenal Datta

**Affiliations:** Department of Aerospace and Mechanical Engineering, University of Notre Dame, Notre Dame, IN 46556, USA; jnajera2@nd.edu (J.N.); mrosenb2@nd.edu (M.R.R.)

**Keywords:** nanomechanical signatures, cancer mechanopathology, viscoelasticity, stiffness, mechanobiomarkers, Young’s modulus

## Abstract

**Simple Summary:**

Atomic force microscopy (AFM) is a powerful technique that has been pivotal to cancer research, but it has only recently been used to study tumor pathology at the tissue scale. In this Review, we highlight studies that have used AFM to characterize the mechanical properties of various cancer tissues and discuss the application of this methodology in the clinic.

**Abstract:**

Atomic force microscopy (AFM) is a popular tool for evaluating the mechanical properties of biological materials (cells and tissues) at high resolution. This technique has become particularly attractive to cancer researchers seeking to bridge the gap between mechanobiology and cancer initiation, progression, and treatment resistance. The majority of AFM studies thus far have been extensively focused on the nanomechanical characterization of cells. However, these approaches fail to capture the complex and heterogeneous nature of a tumor and its host organ. Over the past decade, efforts have been made to characterize the mechanical properties of tumors and tumor-bearing tissues using AFM. This has led to novel insights regarding cancer mechanopathology at the tissue scale. In this Review, we first explain the principles of AFM nanoindentation for the general study of tissue mechanics. We next discuss key considerations when using this technique and preparing tissue samples for analysis. We then examine AFM application in characterizing the mechanical properties of cancer tissues. Finally, we provide an outlook on AFM in the field of cancer mechanobiology and its application in the clinic.

## 1. Introduction

The mechanical exploration of biological materials in the context of disease has gained widespread attention over the past few decades. This has led to the adaptation and development of numerous techniques to study cell and tissue mechanics. These techniques include, but are certainly not limited to, optical tweezers [1], micropipette aspiration [2], parallel-plate rheology [3], and atomic force microscopy (AFM) [4]. AFM in particular has been widely adopted in this field due to its ability to operate in an aqueous environment and at a temperature that most closely mimics the native environment of most biological samples, a feature not offered by other characterization techniques [5,6]. AFM is also superior in its ability to provide high-resolution nanotopographical images and requires relatively simple sample preparation, although special considerations must be made for accurate mechanical analysis [5,6,7,8]. Compared to most other techniques, however, AFM is low-throughput, time-consuming, and technically challenging [6,7,9]. Nonetheless, AFM has led to pioneering work that has advanced our understanding of cell and tissue mechanics, particularly in the context of cancer.

The majority of AFM studies are extensively focused on the nanomechanical characterization of cells, including within the field of cancer [6,7]. Notably, this technique is not only capable of discriminating normal and cancerous cells based on their different mechanical properties [10], but it can also distinguish between varying cancer cell states. For example, when coupled with machine learning and imaging, AFM is able to differentiate two human colon cancer cell lines based on stiffness levels that are inversely proportional to neoplastic aggressiveness (i.e., the more aggressive cell line is less stiff) [11]. Nanotopographical analysis and mechanical characterization via AFM can distinguish which type of regulated cell death process (e.g., intrinsic and extrinsic apoptosis, necrosis, ferroptosis) mouse fibrosarcoma cells are undergoing [12]. More recently, AFM has been used to investigate the effects of cytoskeletal-targeting and non-cytoskeletal-targeting drugs on cancer cell biomechanics, as well as to demonstrate that cancer cells have a softer cortical stiffness than non-malignant cells due to elevated cholesterol in the plasma membrane [13,14,15,16]. In fact, depleting cholesterol using methyl-β-cyclodextrin sensitizes cancer cells to T cell-mediated cytotoxicity in vivo due to enhanced cell–cell contact forces between T cells and stiffened cancer cells [16]. This demonstrates that microenvironmental factors—which play an important role in shaping cancer cell mechanics—can be modulated to promote anti-cancer therapeutic efficacy and immune activity.

These cellular studies have been crucial to better understand cancer mechanopathology. However, in vitro studies typically fail to capture the heterogeneous properties of a tumor and its host organ [17,18]. Instead, tissues represent more relevant samples for studying specific questions in cancer mechanopathology. Indeed, while cancer cells are typically more compliant than their normal counterparts, tumor tissues are usually stiffer due to increased extracellular matrix deposition and cross-linking [6,17]. In fact, increased tissue stiffness is a well-established cancer hallmark that has been linked to tumor progression [19]. This is due in part to the ability of cells to sense and respond to physical cues in the microenvironment via mechanoreception and mechanotransduction. Thus, when cancer cells are subjected to aberrant mechanical conditions present in the tumor microenvironment (e.g., increased stiffness), mechanoresponsive signaling pathways (e.g., YAP/TAZ) are activated to aid in survival and progression under these conditions [20].

It is therefore important that such biomechanical processes and interactions are preserved and studied in situ to develop an accurate understanding of how mechanobiology drives tumorigenesis, metastasis, and treatment resistance. While assays such as immunohistochemistry and immunofluorescence are less technically demanding, AFM’s unique ability to extract mechanical properties from biological samples and relate them to cancer pathogenesis will help reveal novel opportunities for exploration and exploitation in the clinic. Little work has been performed on tumor and tumor-bearing tissues using AFM-based techniques, however, although this approach is gaining traction [6,21].

In this Review, we discuss how AFM is implemented to study tissue mechanics and highlight some of the key challenges associated with this technique. Additionally, we discuss the application of AFM to study cancer mechanopathology at the tissue scale. Finally, we provide an outlook on the use of AFM for future clinical studies and biomarker identification.

## 2. Principles of Atomic Force Microscopy for Studying Tissue Mechanics

### 2.1. General Principles

**Figure 1:**
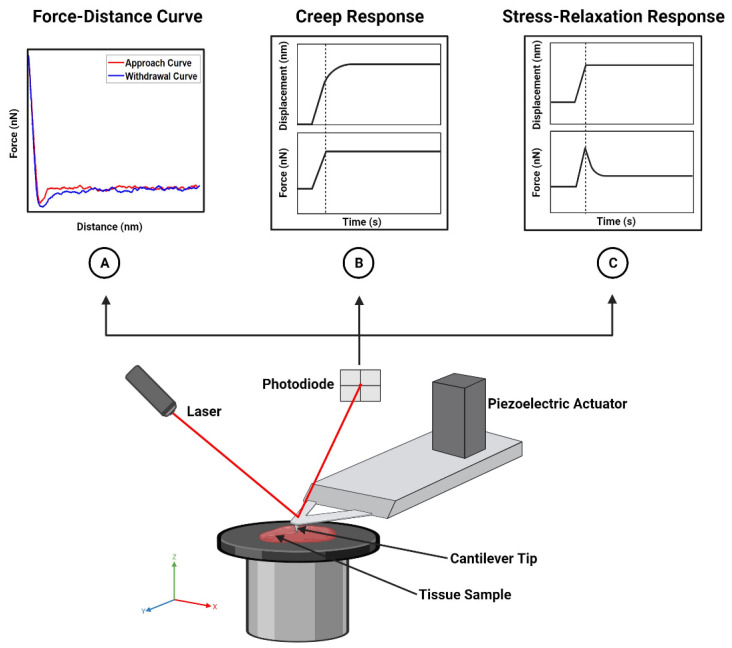
Schematic and application of AFM. The AFM instrument is composed of several parts. The piezoelectric actuator moves the cantilever tip so that it comes into contact with the tissue sample. Upon contact, the tip indents the sample, causing the cantilever to deflect. This deflection is measured by the photodiode. The resulting measurements are used to generate force–distance (**A**), force–time ((**B**,**C**), bottom), or displacement–time curves ((**B**,**C**), top) to characterize the elastic and/or viscoelastic properties of the sample. Created with BioRender.com.

Most tissues are considered soft materials and are therefore unable to bear large magnitudes of stress without being damaged [8,22]. AFM nanoindentation, also known as indentation-type AFM (IT-AFM), has become widely used for determining the mechanical properties of biological samples, including soft tissues [22,23]. The principle of AFM nanoindentation is simple. Briefly, it involves the local indentation/deformation of a biological sample at low loads (typically on the order of nN) [6,22,24]. In order to indent the sample, a probe is brought into contact with the sample via the displacement of a piezoelectric actuator, thereby causing the attached cantilever to deflect, the extent of which is measured by a laser and photodiode (Figure 1). The cantilever deflection is proportional to the applied force, allowing for the construction of a force–displacement curve [25]. AFM tips with a large tip radius (most often spherical and flat-end tips) have the advantage of avoiding high stress concentrations at the point of contact. However, sharper tips, such as pyramidal and conical tips, allow the user to precisely identify where the point of contact occurs in the resulting force–displacement curves [25,26]. It should be noted that in order to obtain accurate nanomechanical measurements using AFM, the inverse optical lever sensitivity (InvOLS) and spring constant of the cantilever must first be calibrated prior to experimentation. The InvOLS is important for converting the signal from voltage to force and is typically calibrated by acquiring force–distance curves on a hard surface [27]. When working with biological samples in liquid, the InvOLS in liquid should be calculated [27]. Spring constant calibration can be performed in a variety of ways, with the most common approaches being the Sader method and global calibration initiative methods [27,28].

It is important to note that force–displacement curves—i.e., curves that are constructed from the deflection data—do not represent true interactions between the tip and sample, whereas force–distance curves (Figure 1A) do [25]. Consequently, well-established contact mechanics models, such as Hertzian, Sneddon, Derjaguin–Muller–Toporov (DMT), and Johnson, Kendall, and Roberts (JKR), are fit to force–distance curves rather than force–displacement curves to appropriately characterize the mechanical properties (e.g., Young’s modulus) of a sample. In practice, after identifying the contact point between the tip and the sample, force–displacement curves are converted to force–distance curves by subtracting the deflection of the cantilever from the displacement of the piezoelectric actuator.

In addition to force–distance curves, force–time curves are used to characterize the viscoelastic properties of a material. This includes measuring the deformation of a sample over time under a constant load (creep response; Figure 1B) or evaluating the time-dependent decrease in stress under constant deformation (stress relaxation response; Figure 1C) [6]. As with force–distance curves, force–time curves are useful for discriminating samples based on their unique viscoelastic profiles. AFM-based stress relaxation tests on chondrocytes isolated from articular cartilage, for example, are able to distinguish superficial chondrocytes (i.e., chondrocytes found in the superficial zone of articular cartilage) from middle/deep chondrocytes (i.e., chondrocytes found in the middle and deep zones of cartilage), as evidenced by their significant differences in the relaxed modulus, Young’s modulus, equilibrium modulus, and apparent viscosity [29].

### 2.2. Models

Hertzian (for spherical indenters) or Sneddon (for conical indenters) contact mechanics models (Equations (1) and (2), respectively) are commonly used to fit force–distance curves and estimate the Young’s modulus of biological samples:

(1) $$\;\;\;F_{Hertz}=\frac{4}{3}\frac{E}{1-\nu^{2}}R^{1/2}\delta^{3/2}$$

(2) $$F_{Sneddon}=\frac{2}{\pi }\frac{E}{1-\nu^{2}}\tan\left(\alpha \right)\delta^{2}$$

where *F* is the applied load; *E* is the Young’s modulus; *R* is the tip radius; ν is the Poisson’s ratio; δ is the indentation depth; and α is the cone half angle. In cases in which the indenter is modified (e.g., a microsphere glued to the free end of a rectangular tip) and/or has a non-spherical and non-conical geometry (e.g., pyramidal), other models are used [30,31,32,33,34]. For example, the equation to analyze curves gathered by a four-sided, blunted pyramidal tip is as follows:

(3) $$F_{Hertzian-Sneddon}=\frac{E}{1-\nu^{2}}\frac{\tan\left(\alpha \right)}{\sqrt{2}}\;\delta^{2}$$

DMT and JKR have been previously used to evaluate the mechanical properties of biological samples, albeit to a lesser extent [6,35,36,37,38]. As with the Hertzian model, the DMT and JKR models can be modified or extended based on the geometry of the indenter [6]. Unlike the Hertz model, however, the DMT and JKR models take into consideration surface forces (e.g., adhesion) outside and inside the contact area, respectively [6,39]. The DMT model is particularly valid for weak surface forces and stiff materials, whereas the JKR model is suitable for compliant materials and strong surface forces [6,39].

The Oliver–Pharr method (Equations (4) and (5)) is another method that is used to calculate the elastic modulus of hard biological materials, such as bone [40], but there are challenges when using this model for soft, viscoelastic tissues [24]:

(4) $$\frac{1}{E_{r}}=\frac{\left(1-\nu_{s}^{2}\right)}{E_{s}}-\frac{\left(1-\nu_{t}^{2}\right)}{E_{t}}$$

(5) $$E_{r}=\frac{\sqrt{\pi }}{2}\frac{S}{\sqrt{A}}\;$$

Here, *E_r_* is the reduced elastic modulus, *s* is the sample, *t* is the tip, *A* is the contact area, and *S* is the stiffness, which is the local slope of the force–distance withdrawal curve at two defined forces (i.e., *S* = ΔF/Δδ). The withdrawal curve is the curve generated as the AFM tip is withdrawn from the sample, while the approach curve is the curve generated as the AFM tip approaches the sample.

Still, fitting AFM data is sometimes challenging and may even yield inconsistent results due to errors in data processing. As reviewed in detail in [41], these errors may be avoided by fitting force–distance curves using the following power law relationship and by considering the indentation depth [41]:

(6) $$F=a\delta^{m}$$

where *a* and *m* are fitting parameters influenced by the tip geometry and mechanical properties of the tissue. The fitting parameter (*m*) is used to calculate the elastic modulus, as follows:

(7) $$F=\frac{1}{m}\frac{2Er_{c}}{1-\nu^{2}}\delta$$

where $r_{c}$ is the contact radius. The contact radius for spherical and pyramidal indenters is given by Equations (8) and (9), respectively:

(8) $$\frac{r_{c}}{R}=c_{1}{\left(\frac{\delta }{R}\right)}^{1/2}+c_{2}\left(\frac{\delta }{R}\right)+c_{3}{\left(\frac{\delta }{R}\right)}^{2}$$

(9) $$r_{c}=\frac{2}{\pi }\left[\delta \tan\left(\alpha \right)+R\left(1-\tan\left(\alpha \right)\right)\right]$$

where $c_{1}$ = 1.0140000, $c_{2}$ = −0.0905900, and $c_{3}$ = −0.0943100.

The Hertz and Sneddon models have helped researchers determine the stiffness of various tissues, including developing brain tissue [42], pulmonary arterial tissue [43], lung tissue [44], mouse heart and pancreatic tissue [22], anterior human corneal tissue [45], and blood vessel tissue [46]. While popular, these models present certain challenges and limitations. Hertzian analysis assumes that the sample is isotropic, homogeneous, linearly elastic, and does not experience large deformations [23,24]. In reality, biological tissues are heterogeneous, anisotropic, and viscoelastic or poroelastic materials [41]. Consequently, a single local measurement is insufficient for accurately developing global tissue mechanical profiles. Instead, multiple local measurements along different regions of the entire tissue sample must be taken to create a mechanical map for that tissue [23,46,47]. It should be noted, however, that for micron-thick tissue samples, data collection is constrained to the surface of the sample. This is because a Hertzian analysis is not applicable for indentation depths that exceed ~10% of a tissue’s thickness. Moreover, the elastic modulus at a single point often varies along the axis of indentation, which thus warrants determining an average elastic modulus instead [41,48,49,50]. Researchers should therefore report their working indentation depths as well as minimize the viscoelastic behavior of their tissue samples if they are using a Hertzian model to quantify the average elastic modulus of their samples [41,49].

### 2.3. Sample Preparation

Along with identifying a suitable tip geometry and contact mechanics model, one must preserve the mechanical properties of the sample during AFM experimentation. Unfortunately, many tissue handling and preparation techniques affect the mechanical characteristics of a sample in one way or another. For example, tissues are often immobilized with an adhesive glue to prevent them from moving during data collection [8,26]. Although there are ways to avoid direct contact between the adhesive and tissue, chemicals diffusing from the glue can have an effect on the mechanical properties of the specimen [8].

Chemically fixing and cryosectioning tissues is another way tissues lose their mechanical integrity. Chemical fixation is a common method that uses fixatives such as formaldehyde or glutaraldehyde to preserve the tissue microarchitecture by cross-linking proteins and halting biochemical processes [51]. As a result, this causes tissue hardening [51,52]. Cryosectioning is a technique that involves freezing the tissue and cutting it into thin slices using a cryostat [53]. This is desirable, as it is otherwise difficult to cut tissues into exact dimensions [54]. However, the freezing process often causes tissue damage and stiffening via ice crystal formation and cell death [8]. Because of these drawbacks, fixed and cryosectioned tissues are mostly used for imaging rather than mechanical characterization [8,55]. Noise that arises from the cantilever tip electrostatically interacting with the biological sample and its medium also poses a challenge [26].

**Figure 2:**
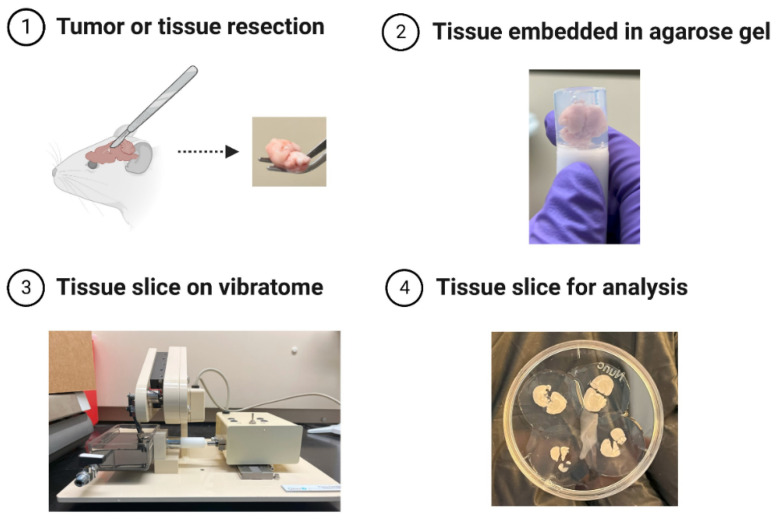
Sectioning Brain Tissue for AFM Analysis. Live brain tissue is embedded in an agarose matrix to preserve its mechanical properties and viability. A vibratome is then used to create thin slices of the live brain embedded in agarose, which are used for further analysis by AFM (Panel 1 mouse image was created with BioRender.com).

**Figure 3:**
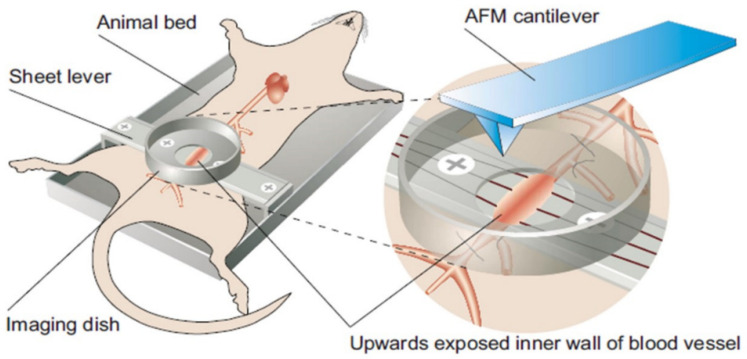
In Vivo Nanomechanical Characterization of Vessels Using AFM. A rat with a hollow dish over the aortic intima exposes the vessel to the AFM tip for nanomechanical characterization. Reprinted/adapted with permission from Ref. [46]. 2009, AIP Publishing.

A few approaches have been designed to circumvent some of these problems. Farnier et al., for example, use a vibratome to cut thin slices of live brain tissue embedded in an agarose matrix (Figure 2). This method avoids the need for chemical fixation, preserves the mechanical properties of the tissue because the agarose does not infiltrate the tissue, and reduces the likelihood that the slice will be physically damaged (e.g., torn) [55]. In another study by Mao et al. [46], the authors repurpose the atomic force microscope for in vivo nanomechanical imaging and characterization in rats. In this study, a three-component surgical platform that contains a hollow dish is used to expose the aortic intima for easy accessibility by the cantilever tip (Figure 3). This approach has the benefit of mechanically characterizing vessels in their in vivo native state. In studies with human cancer tissue biopsies, the resected tissue is sometimes preserved in a relevant buffer or medium containing specific enzyme inhibitors (e.g., protease and phosphatase inhibitors), immobilized on a glass slide, and then immediately measured [17,56].

## 3. Application of Atomic Force Microscopy to Study Cancer Pathology

Alterations in the mechanical properties of cells and tissues provide valuable information about cancer pathology. Many AFM studies focus extensively on the nanomechanical characterization of cells to establish novel mechanical biomarkers. These “mechanobiomarkers” may provide insight into the progression of the disease, enhance cancer detection, as well as improve therapeutic strategies [57,58,59]. However, in addition to irreproducible measurements between different groups [41], clinicians are reluctant to adopt AFM-based single-cell nanomechanical characterization as a diagnostic tool because it often fails to capture the complexities of a tumor and the organ in which it is situated [18]. Thus, mechanically characterizing the tumor and surrounding normal tissue along with single-cell measurements may be more relevant for clinical application.

**Figure 4:**
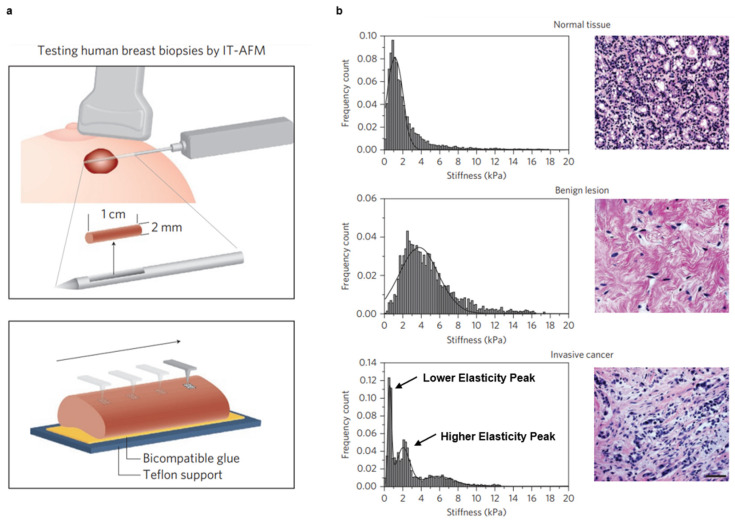
Creating a Nanomechanical Signature for Breast Cancer Using IT-AFM. (**a**) Schematic of IT-AFM collecting local deformation measurements along a human breast biopsy that is immobilized on a glass slide. (**b**) Stiffness profiles for normal, benign, and malignant human breast tissue. Normal and benign tissues have a unimodal stiffness distribution, while malignant tissues are characterized by a bimodal distribution. The first peak, or lower elasticity peak, corresponds to the softer cancer cells in the tumor core, while the second peak, or higher elasticity peak, represents the stiffer stroma at the tumor periphery. Scale bar for all histological images is 50 μm. Reprinted/adapted with permission from Ref. [17]. 2012, Springer Nature.

Over the past decade, efforts have been made to develop nanomechanical signatures for various cancers. In 2012, Plodinec et al. used AFM (Figure 4a) to create stiffness profiles of normal, benign, and malignant human breast biopsy tissue [17]. Notably, the stiffness profiles of normal and benign human breast tissues are unimodal, whereas malignant tissues are bimodal (Figure 4b). The lack of uniformity observed for the latter stems from the heterogeneous nature of the tissues, wherein one peak (i.e., the lower elasticity peak) of the profile corresponds to soft cancer cells while the other (i.e., the higher elasticity peak) corresponds to the stiffer tumor stroma surrounding these cells. Similar results were seen throughout various stages of breast cancer progression in an MMTV-PyMT spontaneous mouse model [17].

In 2015, Tian et al. performed a similar study on ex vivo human liver tissue (normal, cirrhotic, primary liver cancer, and recurrent liver cancer) to improve the diagnosis of hepatocellular carcinoma [56]. The elasticity maps of the liver tissues in this study once again show that cancer cells represent a lower elasticity peak, whereas the extracellular matrix represent a higher elasticity peak. The distribution of higher elasticity peaks is extremely variable, however. As a result, the unique nanomechanical signatures of the liver tissues during different stages of cancer progression are made up of lower-elasticity-peak data. Notably, the authors found that changes in mechanical properties differentiate liver cancer tissues from cirrhotic and normal liver tissues and may even predict tumor recurrence following treatment [56].

The following year, Ciasca et al. used AFM to develop nanomechanical signatures of malignant glioblastoma and benign meningothelial meningioma brain tumors [60]. Similar to the previous two studies, they created apparent Young’s modulus maps of glioblastoma tumors along necrotic and non-necrotic regions. Non-necrotic tissues possess two distinct peaks corresponding to the presence of soft and stiff structures, while necrotic tissues are uniformly distributed owing to the increased activity of extracellular-matrix-degrading enzymes, such as matrix metalloproteinase [60]. These distinct properties may serve as biomechanical signatures for classifying glioblastoma progression. Meningothelial meningioma tissues, in contrast, are stiffer than glioblastoma and normal brain tissues and have defined peaks in their stiffness profiles [60].

Most recently, Stylianou et al. characterized the distinct nanomechanical properties of healthy pancreas tissue and pancreatic tumors over various stages of progression [61]. Unsurprisingly, the Young’s modulus increases as the cancer progresses, and the elastic distribution of normal pancreas tissue has a single peak, whereas pancreatic tumors have distinct lower elasticity peaks and higher elasticity peaks that relate to cancer cell softening and desmoplasia, respectively [61]. By combining AFM with polarized light microscopy of picrosirius red-stained tissues, the authors also show that the higher elastic values are due to measurements collected in collagen-rich areas of the tissue. These findings open the possibility of developing novel mechanobiomarkers [61]. AFM has also been previously combined with other microscopy techniques to study cells and tissues, such as optical microscopy, second-harmonic generation, and scanning electron microscopy, as reviewed in ref. [62].

Unlike most AFM studies, Ciasca et al. also investigated the viscoelastic response of brain cancer tissues. To evaluate this response, hysteresis was used as a proxy for viscous effects [60]. Hysteresis is quantified by calculating the difference in the area under the approach curve and area under the withdrawal curve normalized to the area under the approach curve. With this approach, they demonstrated that viscous forces are stronger in necrotic glioblastoma and meningothelial meningioma tumors than in non-necrotic glioblastoma tumor tissue and dura tissue infiltrated with neoplastic cells [60]. Indeed, viscous forces are important in other contexts as well. One study found that while the elastic modulus alone is able to differentiate between breast, kidney, and thyroid cancer subtypes, viscosity is able to discriminate between normal and malignant thyroid tissue [30]. Here, the elastic modulus and viscosity are quantified using Equation (3) and the creep function for a standard linear solid viscoelastic model, respectively. In a similar study involving human prostate tumor tissues, Tang et al. found that tumors tend to be more compliant and less viscous the more abnormal the cancer cells look [31].

Another study that investigated breast cancer bone metastases found that the elastic modulus and viscosity of the metastatic tumor is extremely low and influenced by the metastatic niche in the bone [33]. The elastic modulus is quantified in two ways: (1) by fitting force–distance curves to the Hertz–Sneddon model (*E_H-S_*), and (2) by fitting creep curves to a Kelvin–Voigt model (*E_K-V_*). The latter approach is also used to determine the viscosity (*η*) [33,34]. Interestingly, the *E_H-S_*, E_K-V_, and η do not show statistically significant differences between tumor-bearing and non-tumor-bearing bone, thus suggesting that the mechanical properties of the surrounding bone microenvironment are not altered in the presence of the metastatic tumor [33].

Atomic force microscopy is also able to monitor the effect of drug treatment on the nanomechanical properties of solid tumors [21]. Stylianou et al. found that combining tranilast (an antihistamine) with doxorubicin (a chemotherapy) to treat HT1080 fibrosarcoma and E0771 breast cancer murine models significantly reduces the Young’s modulus and greatly improves the chemotherapeutic efficacy. Importantly, tranilast is a type of “mechanotherapeutic” that is able to reduce tumor stiffness by reducing the levels of extracellular components, namely, collagen and hyaluronan [21]. Therefore, AFM is capable of establishing mechanobiomarkers that evolve with tumor nanomechanical property alterations over the course of therapeutic intervention. While AFM has been previously used to study the effect of treatment on cancer cells [7,63,64], its application at the tissue scale is relatively new. Therefore, evaluating the impact of mechanotherapeutics on cancer tissue mechanics presents an exciting opportunity for researchers to further explore.

## 4. Conclusions and Future Directions

Changes in the mechanical properties of cells and tissues over the course of cancer development may provide valuable insight for the design of novel therapeutic strategies and improve the diagnosis and staging of various cancers. Owing to its ability to study biological materials in physiologically relevant conditions, AFM has become a popular method for evaluating cell and tissue mechanics [5,6]. Recognizing the importance of whole-tissue mechanics in cancer progression, many studies are now beginning to use AFM to create nanomechanical signatures of various malignant and non-malignant tissues (Table 1). Most of the nanomechanical signatures described in this Review are largely based on using the Hertzian, Sneddon, or a similar contact mechanics model to construct Young’s modulus maps of tissue samples. As a result, many tumor and tumor-bearing tissues are now known to possess a distinct mechanical fingerprint relative to healthy tissue.

The mechanical identity of a biological material is not simply defined by its elastic properties. Indeed, researchers are beginning to explore the relevance of viscous forces in their tissue specimens. Considering such viscous forces will help in identifying novel mechanobiomarkers and developing a more complete mechanical profile of a diseased tissue. Future studies should therefore focus on characterizing the viscoelastic behavior of tissues to enhance our understanding of cancer tissue mechanopathology and improve diagnosis and therapy in the clinic. However, as this is a relatively new application of AFM to biological materials, criteria for viscoelastic characterization are not well established; parameters such as the indentation depth and rate, along with the viscoelastic model used, should be optimized in these studies.

With sophisticated computational approaches slowly gaining traction in AFM studies, errors in data processing may become less prevalent and reduce the time spent analyzing data. Notably, finite and inverse finite element models [47,65,66] and machine learning algorithms [9,67,68,69] have been developed and implemented to analyze AFM nanoindentation data more accurately. Minelli et al., for example, were able to discriminate healthy tissues from cancer tissues using a fully automated neural network analysis that evaluates force–distance curves [69]. The automation of AFM data analysis may likely prove to be very valuable in the clinic. However, a unified approach for studying tissue specimens with this technique is lacking. Efforts should be made to standardize the application of AFM to achieve repeatable and accurate results before considering clinical translation. This includes working with consistent operating parameters across different samples (e.g., indentation depth, indentation rate, scanning area, force), using the same tip properties, being consistent with sample handling and treatment, applying appropriate models for mechanical characterization, and comparing results with other classical material characterization techniques for verification [7]. These considerations may vary between different types of samples, thereby necessitating optimization experiments to identify the most suitable parameters for a particular sample type. Studies such as those presented in this Review lay the groundwork for identifying optimal procedures for the AFM-based material and mechanical characterization of cancerous tissues. Additionally, generalized approaches, such as those presented in [23,41], may be more suitable for an accurate and standardized mechanical characterization of tissue samples, as they are not limited by the same constraints posed by the Hertzian and Sneddon models. Such standardization could enhance the impact and applicability of automated data analysis. Altogether, by taking these considerations into account, AFM has the capacity to serve as a powerful tool in cancer research, with clinically relevant applications, including the identification and validation of treatment-sensitive mechanobiomarkers.

## Figures and Tables

**Table 1:** List of studies evaluating the mechanical properties of tumor, tumor-bearing, and non-tumor-bearing tissues using AFM.

Year	Sample	Property	Model/Method	Author	Reference
2012	Normal, benign, and malignant breast tissue	Young’s Modulus	Oliver–Pharr	Plodinec et al.	[17]
2015	Normal liver tissue; cirrhotic, primary, and recurrent liver cancer tissue ^1^	Young’s Modulus	Sneddon	Tian et al.	[56]
2016	Normal brain tissue; glioblastoma (necrotic and non-necrotic) and meningothelial meningioma brain tumor tissue	Young’s Modulus	Sneddon	Ciasca et al.	[60]
		Hysteresis	$\frac{A_{E}-A_{R}}{A_{E}}$ ^2^		
2019	Prostate tumor tissue	Young’s Modulus	Hertzian–Sneddon ^3^	Tang et al.	[31]
		Viscosity	N/A		
2021	Breast cancer bone metastases, bone metaphysis region (with and without tumor)	Young’s Modulus	Hertzian–Sneddon ^4^ Kelvin–Voigt	Chen et al.	[33]
		Viscosity	Kelvin–Voigt		
2022	Normal breast, kidney, and thyroid tissue; breast, kidney, and thyroid tumor tissue	Young’s Modulus	Hertzian–Sneddon ^3^	Levillain et al.	[30]
		Viscosity	Standard Linear Solid		
2022	Breast and fibrosarcoma tumors	Young’s Modulus	Hertzian	Stylianou et al.	[21]
2023	Normal pancreatic tissue and pancreatic tumor tissue	Young’s Modulus	Hertzian	Stylianou et al.	[61]

^1^ In addition to liver tissue, this study also looked at 1 renal cell carcinoma specimen, 1 esophageal cancer specimen, and 1 colon cancer specimen for comparison. ^2^ *A_E_*: area under approach curve, *A_R_*: area under withdrawal curve. ^3^ This study uses the Hertzian–Sneddon model for a pyramidal indenter (i.e., Equation (3)). ^4^ A Taylor’s series expansion was performed on the Hertzian–Sneddon model for a rectangular indenter with a microsphere glued to the free end. See [34], Supplemental Information, for more information.
